# Supplementary material for: Cryo-EM structures reveal tau filaments from Down syndrome adopt Alzheimer’s disease fold
Source: Acta Neuropathol Commun. 2024 Jun 12;12:94. doi: 10.1186/s40478-024-01806-y (PMC11167798; doi:10.1186/s40478-024-01806-y)
Supplement: Supplementary file 2 — Additional file 2. [file 40478_2024_1806_MOESM2_ESM.docx]

| **Brain bank** | **Case ID** | **Case # in study** | **Age at death** | **Sex** | **APOE genotype** | **Clinical status** | **Brain region** | **Postmortem interval (h)** | **Approx brain mass (g)** | **Tau prion activity (DxA)^*^** | **Insoluble total tau (µg/g); formic acid extract^#^** | **Xtau neuropath score^#^** | **Braak stage (NFTs)** | **Aβ prion activity (DxA)^*^** | **Insoluble Aβ (µg/g); formic acid extract^#^** | **XAβ neuropath score^#^** | **CERAD score**  **(amyloid plaques)** |
| --- | --- | --- | --- | --- | --- | --- | --- | --- | --- | --- | --- | --- | --- | --- | --- | --- | --- |
| BCN | **907** | **1** | 63 | M | ε3/ε3 | Dementia documented | Frontal cortex | 6 | **1.192** | 253341.25 | 23618 | 4 | VI | 185071.2 | 108 | 3 | C3 |
| UMD | **4870** | **2** | 51 | F | ε2/ε3 | Dementia documented | Frontal cortex | 4 | **1.025** | 179783.64 | 34341 | 4 | ND | 136564.9 | 67 | 4 | ND |
| UMD | **4659** | **3** | 46 | F | ε3/ε4 | No dementia documented | Frontal cortex | 7 | **0.791** | 257720.75 | 102623 | 4 | ND | 170006.7 | 109 | 4 | ND |
| BCN | **714** | **4** | 36 | F | ε3/ε3 | No dementia documented | Frontal cortex | 12 | **1.383** | 172328.76 | 20022 | 2 | II | 100221.7 | 41 | 2 | C2 |

**Supplementary Table S1.** Fresh-frozen postmortem donor tissues used for filament extraction and cryo–electron microscopy.

Abbreviations: Aβ, amyloid-β; APOE, apolipoprotein E; BCN, Barcelona; CERAD, Consortium to Establish a Registry for Alzheimer's Disease; DxA, density × area; F, female; M, male; ND, not determined; NFT, neurofibrillary tangle; UMD, University of Maryland; Xtau, custom measurement of immunostained neurofibrillary tau tangles in fixed medial frontal cortex slices; XAβ, custom measurement of immunostained Aβ plaques in fixed medial frontal cortex slices. *We previously generated the Tau and Aβ prion activity measurements in Condello et al. (DOI: 10.1073/pnas.2212954119). ^#^We previously generated the Xtau and XAβ scores in Maxwell et al. (DOI: 10.1186/s40478-021-01298-0).

| **Brain bank** | **ID** | **Disease** | **Region** | **UMAP name** | **Age at death** | **Sex** | **APOE** | **Postmortem interval (h)** | **Xtau neuropath score** | **XAβ neuropath score** |
| --- | --- | --- | --- | --- | --- | --- | --- | --- | --- | --- |
| KCL | A035/03 | AD | Frontal cortex | AD 1 | 59 | F | ND | 36 | 4 | 4 |
| KCL | A061/03 | AD | Frontal cortex | AD 2 | 55 | M | ND | 18 | 4 | 4 |
| OXF | NP177-2013 | AD | Frontal cortex | AD 3 | 90 | F | ε3/ε3 | 48 | 4 | 3 |
| UCI | 14-08 | AD | Frontal cortex | AD 4 | 86 | M | ε3/ε3 | 4 | 2 | 2 |
| UCI | 37-15 | AD | Frontal cortex | AD 5 | 87 | F | ε3/ε4 | 4 | 2 | 3 |
| UCI | 4-02 | AD | Frontal cortex | AD 6 | 83 | M | ε3/ε4 | 3 | 4 | 3 |
| BCN | 907 | DS | Frontal cortex | DS 1 | 63 | M | ε3/ε3 | 6 | 4 | 3 |
| BCN | 714 | DS | Frontal cortex | DS 2 | 36 | F | ε3/ε3 | 12 | 2 | 2 |
| UMD | 4335 | DS | Frontal cortex | DS 3 | 28 | M | ε4/ε4 | 26 | 2 | 2 |
| UMD | 4870 | DS | Frontal cortex | DS 4 | 51 | F | ε2/ε3 | 4 | 4 | 4 |
| UMD | 5510 | DS | Frontal cortex | DS 5 | 65 | M | ε3/ε3 | 10 | 4 | 4 |
| UMD | 5600 | DS | Frontal cortex | DS 6 | 57 | M | ε3/ε3 | 6 | 4 | 3 |
| UCI | 29-06 | DS | Frontal cortex | - | 45 | F | ε3/ε3 | 3 | 4 | 4 |
| UCI | 29-06 | DS | Temporal cortex | - | 45 | F | ε3/ε3 | 3 | - | - |
| UCI | 3-17 | DS | Frontal cortex | - | 57 | M | ε3/ε3 | 4 | 3 | 4 |
| UCI | 3-17 | DS | Temporal cortex | - | 57 | M | ε3/ε3 | 4 | - | - |
| UCI | 30-00 | DS | Frontal cortex | - | 61 | M | ε3/ε3 | 11 | 4 | 4 |
| UCI | 30-00 | DS | Temporal cortex | - | 61 | M | ε3/ε3 | 11 | - | - |
| UCI | 46-94 | DS | Frontal cortex | - | 62 | F | ε3/ε3 | 3 | 4 | 4 |
| UCI | 46-94 | DS | Temporal cortex | - | 62 | F | ε3/ε3 | 3 | - | - |
| UCI | 30-05 | DS | Frontal cortex | - | 57 | F | ε3/ε3 | 3 | 4 | 4 |
| UCI | 30-05 | DS | Temporal cortex | - | 57 | F | ε3/ε3 | 3 | - | - |

**Supplementary Table S2.** Fixed postmortem donor tissues used for EMBER analysis.

Abbreviations: Aβ, amyloid-β; AD, Alzheimer’s disease; APOE, apolipoprotein E; BCN, Barcelona; DS, Down syndrome; EMBER, excitation multiplexed bright emission recording; F, female; KCL, King’s College London; M, male; ND, not determined; OXF, Oxford; UCI, University of California, Irvine; UMAP, uniform manifold approximation and projection; UMD, University of Maryland; Xtau, custom measurement of immunostained neurofibrillary tau tangles in fixed medial frontal cortex slices; XAβ, custom measurement of immunostained Aβ plaques in fixed medial frontal cortex slices. We generated the Xtau and XAβ scores in Maxwell et al. (doi.org/10.1186/s40478-021-01298-0); (-), not determined.

| **Brain** |  |  |  |  |  |  | **HTRF Tau - Sarksosyl Insoluble** | | | **HTRF Tau - Sarkosyl Soluble** | | |
| --- | --- | --- | --- | --- | --- | --- | --- | --- | --- | --- | --- | --- |
| **bank** | **Case ID** | **Cohort** | **Age** | **Sex** | **PMI (h)** | **APOE** | **Replicate 1** | **Replicate 2** | **Mean** | **Replicate 1** | **Replicate 2** | **Mean** |
| UMD | 5277 | DS | 19 | M | 26 | 3/4 | 15645.1 | 16458.7 | 16051.9 | 603.1 | 597.5 | 600.3 |
| MIA | 18 | DS | 24 | M | 24 | 3/3 | 545.8 | 542.8 | 544.3 | 542.9 | 542.5 | 542.7 |
| UMD | 5341 | DS | 25 | M | 24 | 3/3 | 553.0 | 548.9 | 550.9 | 545.3 | 545.8 | 545.5 |
| UMD | 4335 | DS | 28 | M | 26 | 4/4 | 2568.1 | 2076.7 | 2322.4 | 554.8 | 559.3 | 557.1 |
| BCN | 714 | DS | 36 | F | 12 | 3/3 | 2166.6 | 5923.8 | 4045.2 | 563.9 | 565.5 | 564.7 |
| UMD | 4904 | DS | 40 | M | 10 | 3/3 | 4267.3 | 4013.1 | 4140.2 | 562.6 | 570.8 | 566.7 |
| UMD | 5783 | DS | 41 | M | 7 | 3/4 | 554.8 | 556.3 | 555.6 | 546.4 | 545.8 | 546.1 |
| UMD | 4659 | DS | 46 | F | 7 | 3/4 | 17361.5 | 15368.8 | 16365.2 | 891.5 | 877.5 | 884.5 |
| UCI | 7-17 | DS | 47 | F | 7 | 3/3 | 7634.3 | 7636.4 | 7635.3 | 2239.4 | 1583.0 | 1911.2 |
| UCI | 32-15 | DS | 49 | M | 6 | 3/3 | 10617.7 | 8329.6 | 9473.7 | 2670.9 | 2536.6 | 2603.7 |
| UMD | 4870 | DS | 51 | F | 4 | 2/3 | 17225.9 | 14728.8 | 15977.4 | 798.9 | 784.0 | 791.4 |
| UCI | 3-17 | DS | 57 | M | 4 | 3/3 | 6796.5 | 7600.6 | 7198.5 | 2059.0 | 1441.0 | 1750.0 |
| UMD | 5600 | DS | 57 | F | 6 | 3/3 | 12901.8 | 15353.2 | 14127.5 | 1162.8 | 1222.3 | 1192.6 |
| UMD | 6151 | DS | 57 | M | 5 | 3/3 | 11733.7 | 8897.7 | 10315.7 | 1796.3 | 1735.5 | 1765.9 |
| UCI | 39-17 | DS | 58 | M | 6 | 3/4 | 13579.0 | 12312.8 | 12945.9 | 1640.6 | 1673.0 | 1656.8 |
| BCN | 1335 | DS | 62 | F | 9 | 3/4 | 5821.2 | 5543.6 | 5682.4 | 1279.2 | 1457.8 | 1368.5 |
| BCN | 1469 | DS | 62 | M | 6 | 3/4 | 16991.4 | 14799.6 | 15895.5 | 709.0 | 699.1 | 704.0 |
| BCN | 907 | DS | 63 | M | 6 | 3/3 | 18942.7 | 17465.2 | 18204.0 | 1065.7 | 1002.4 | 1034.1 |
| UMD | 5386 | DS | 64 | M | 20 | 3/4 | 9503.0 | 8037.1 | 8770.0 | 627.8 | 619.4 | 623.6 |
| UMD | 5510 | DS | 65 | M | 10 | 3/3 | 17315.0 | 14110.2 | 15712.6 | 658.8 | 665.9 | 662.3 |
| UCI | 4-16 | DS | 70 | M | 4 | 3/3 | 6422.3 | 6764.8 | 6593.6 | 1899.6 | 1370.1 | 1634.9 |
| UCI | 21-06 | AD | 82 | M | 5 | 3/4 | 1594.2 | 1561.5 | 1577.9 | 554.0 | 551.8 | 552.9 |
| UCI | 04-02 | AD | 83 | M | 3 | 3/4 | 15727.8 | 17243.0 | 16485.4 | 1100.9 | 1023.3 | 1062.1 |
| UCI | 14-08 | AD | 86 | M | 4 | 3/3 | 17272.3 | 17205.9 | 17239.1 | 719.3 | 685.2 | 702.3 |
| UCI | 37-15 | AD | 87 | F | 4 | 3/4 | 14572.5 | 16351.5 | 15462.0 | 850.5 | 828.6 | 839.6 |
| UCI | 37-15 | AD | 87 | F | 4 | 3/4 | 19684.5 | 20442.3 | 20063.4 | 776.5 | 695.3 | 735.9 |
| UCI | 10-17 | ADNC | 66 | F | 4 | 3/4 | 3615.8 | 2116.9 | 2866.4 | 598.9 | 594.3 | 596.6 |
| UCI | 46-16 | ADNC | 78 | M | 3 | 3/4 | 2028.3 | 1590.5 | 1809.4 | 562.6 | 555.8 | 559.2 |
| BCN | 1937 | ADNC | 83 | F | 8 | 3/3 | 1921.2 | 1603.3 | 1762.2 | 550.5 | 553.6 | 552.1 |
| BCN | 1858 | ADNC | 83 | F | 8 | 3/3 | 14397.1 | 8150.2 | 11273.7 | 570.4 | 604.3 | 587.4 |
| UCI | 18-08 | ADNC | 84 | F | 4 | 3/3 | 606.2 | 619.9 | 613.0 | 548.2 | 549.8 | 549.0 |
| BCN | 1949 | ADNC | 86 | M | 8 | 3/3 | 601.7 | 586.4 | 594.0 | 539.7 | 545.7 | 542.7 |
| UCI | 14-17 | ADNC | 89 | F | 6 | 2/3 | 4252.2 | 4536.0 | 4394.1 | 585.4 | 588.7 | 587.0 |
| BCN | 1870 | ADNC | 97 | F | 7 | 3/3 | 749.4 | 774.4 | 761.9 | 545.5 | 550.4 | 547.9 |

**Supplementary Table S3.** Fresh-frozen postmortem donor tissues used for HTRF and Western blot analysis.

Abbreviations: AD, Alzheimer’s disease; ADNC, Alzheimer’s disease neuropathological change; APOE, apolipoprotein E; BCN, Barcelona; DS, Down syndrome; F, female; HTRF, Homogenous Time-Resolved Fluorescence Assay; M, male; MIA, University of Miami; PMI, post-mortem interval in hours; UCI, University of California, Irvine; UMD, University of Maryland.

|  | Case 1 | | Case 2 | | Case 3 | | Case 4 | |
| --- | --- | --- | --- | --- | --- | --- | --- | --- |
| **Data collection** | PHFs | SFs | PHFs | SFs | PHFs | SFs | PHFs | CTE |
| Magnification | x105,000 | x105,000 | x105,000 | x105,000 | x105,000 | x105,000 | x105,000 | x105,000 |
| Defocus range (mm) | -0.8 to -1.8 | -0.8 to -1.8 | -0.8 to -1.8 | -0.8 to -1.8 | -0.8 to -1.8 | -0.8 to -1.8 | -0.8 to -1.8 | -0.8 to -1.8 |
| Voltage (kV) | 300 | 300 | 300 | 300 | 300 | 300 | 300 | 300 |
| Microscope | Titan Krios | Titan Krios | Titan Krios | Titan Krios | Titan Krios | Titan Krios | Titan Krios | Titan Krios |
| Detector | Gatan K3 | Gatan K3 | Gatan K3 | Gatan K3 | Gatan K3 | Gatan K3 | Gatan K3 | Gatan K3 |
| Frame exposure time (s) | 2.024 | 2.024 | 2.024 | 2.024 | 2.024 | 2.024 | 2.024 | 2.024 |
| Dose rate (e-/physical pixel/sec) | 16 | 16 | 16 | 16 | 16 | 16 | 16 | 16 |
| Total dose (e-/Å-2) | 46 | 46 | 46 | 46 | 46 | 46 | 46 | 46 |
| Pixel size (Å) | 0.834 | 0.834 | 0.834 | 0.834 | 0.834 | 0.834 | 0.834 | 0.834 |
| Movies collected | 9160 | | 5902 | | 6949 | | 1293 | |
| **Grids & sample** | Conventional grids Pronase | | Affinity grid no Pronase | | Conventional grid no Pronase | | Affinity grid no Pronase | |
| **Reconstruction** | | | | | | | | |
| Box size (pixel) | 280 | 280 | 280 | 280 | 280 | 280 | 280 | 280 |
| Inter-box distance (Å) | 17 | 17 | 17 | 17 | 17 | 17 | 17 | 17 |
| Total segments* | 1127462 | 329537 | 7488898 | 287943 | 271767 | 86388 | 197928 | 17473 |
| Final Particles (no.) | 215640 | 47801 | 79599 | 26964 | 47264 | 35177 | 32831 | N/A** |
| Resolution (Å) | 2.7 | 3 | 3.1 | 3.2 | 2.9 | 3.1 | 5 | 7.8 |
| B-factor (Å2) | -83.71 | -81.34 | -89.69 | -71.29 | -68.79 | -90.05 | -262.95 | -415.37 |
| Helical rise (Å) | 2.39 | 4.81 | 2.37 | 4.77 | 2.4 | 4.81 | 2.38 | 2.38 |
| Helical twist (°) | 179.45 | -1.08 | 179.5 | -1.04 | 179.48 | -1.06 | 179.45 | 179.55 |

*after initial 2D classification

**no subsequent classificaiton

**Supplementary Table S4.** Cryo–electron microscopy data collection and structure determination.

Abbreviations: CTE, chronic traumatic encephalopathy; N/A, not applicable; PHF, paired helical filament; SF, straight filament.

|  | Case 1 | | Case 2 | |
| --- | --- | --- | --- | --- |
| **Model** | PHFs | SFs | PHFs | SFs |
| model resolution | 2.7 | 3.0 | 3.1 | 3.5 |
| atoms | 35910 | 39501 | 40698 | 39513 |
| Residues | 2310 | 2541 | 2618 | 2541 |
| Bonds (RMSD) |  |  |  |  |
| Length (Å) (# > 4σ) | 0.010 (0) | 0.010 (0) | 0.010 (0) | 0.011 (0) |
| angles (°) (# > 4σ) | 1.717 (92) | 1.791 (70) | 1.871 (38) | 1.914 (95) |
| MolProbity score | 2.21 | 2.32 | 2.53 | 2.63 |
| Clash score | 18.87 | 32.66 | 40.97 | 46.84 |
| Ramachandran plot (%) |  |  |  |  |
| Outliers | 0.00 | 0.00 | 0.00 | 1.33 |
| Allowed | 6.67 | 4.69 | 6.67 | 6.67 |
| Favored | 93.33 | 95.31 | 93.33 | 92 |
| Rotamer outliers (%) | 0.00 | 0.00 | 0.00 | 0.00 |

**Supplementary Table S5.** Cryo–electron microscopy model building.

Abbreviations: #, number; PHF, paired helical filament; RMSD, root mean square deviation; SF, straight filament.
