# Supplementary material for: Cryo-EM structures reveal tau filaments from Down syndrome adopt Alzheimer’s disease fold
Source: Acta Neuropathol Commun. 2024 Jun 12;12:94. doi: 10.1186/s40478-024-01806-y (PMC11167798; doi:10.1186/s40478-024-01806-y)
Supplement: Supplementary file 1 — Additional file 1. [file 40478_2024_1806_MOESM1_ESM.docx]

**SUPPLEMENTARY INFORMATION**

**Cryo-EM Structures Reveal Tau Filaments from Down Syndrome Adopt Alzheimer’s Disease Fold**

**AUTHORS:** Ujjayini Ghosh^1†^, Eric Tse^1†^, Hyunjun Yang^1^, Marie Shi^1^, Christoffer D. Caro^1^, Feng Wang^2^, Gregory E. Merz^1,3^, Stanley B. Prusiner^1,2,3^, Daniel R. Southworth^1,2^*, Carlo Condello^1,3^*

**AFFILIATIONS:**

^1^Institute for Neurodegenerative Diseases, University of California San Francisco, San Francisco, CA, USA

^2^Department of Biochemistry and Biophysics, University of California San Francisco, San Francisco, CA, USA

^3^Department of Neurology, University of California San Francisco, San Francisco, CA, USA

†Equal Contribution

*Correspondence: [daniel.southworth@ucsf.edu](mailto:daniel.southworth@ucsf.edu); [carlo.condello@ucsf.edu](mailto:carlo.condello@ucsf.edu)

**KEY WORDS:** Down syndrome, Alzheimer’s disease, tauopathy, protein conformation, cryo–electron microscopy

**Supplementary Figure S1**. Fluorescence images of immunolabeled Aβ42 and Aβ40 plaques in DS brain samples used for cryo–electron microscopy experiments. Formalin-fixed frontal cortex sections stained with Aβ isotype-specific antibodies against C-terminal-length isoforms Aβ42 (cyan) and Aβ40 (orange) in DS case 4, 36 years old (A-C), DS case 3, 46 years old (D-F), and DS case 2, 51 years old (G-I). Individual fluorescence channels were converted to grayscale and inverted for Aβ42 (B, E, H) and Aβ40 (C, F, I). Scale bar = 2 mm. Inset dimensions = 0.6 mm^2^.

**Supplementary Figure S2.** Biochemical characterization of sarkosyl-insoluble tau from DS and AD frontal cortex. (A and B) Quantification of aggregated tau in sarkosyl-soluble and sarkosyl-insoluble extracts from frozen frontal cortex of DS (<40 years old and >40 years old), AD, and ADNC (Alzheimer’s disease neuropathologic change; individuals with no cognitive impairment but have presence of modest AD neuropathology). HTRF assay positive control and negative control values are represented by the upper and lower dotted y-axis lines, respectively. HTRF Tau aggregation assay shows only marginal aggregated tau in the sarkosyl-soluble fraction for DS (<40 yo), AD, and ADNC with the exception of DS (>40 yo) group showing modest levels of aggregated tau for some samples (A). HTRF Tau aggregation assay shows robust levels of aggregated tau in the sarkosyl-insoluble fraction for most of the DS (>40 yo) and AD samples; modest levels of aggregated tau were detected for a subset of DS (<40 yo) and ADNC samples (B). Based on the HTRF Tau aggregation values in the sarkosyl-insoluble fraction, we selected a subset of samples for Western blot as indicated by the numbers on panel B. (C–F). Western blot analysis of undigested and trypsin-digested sarkosyl-insoluble tau. (C and D) T13 and R4 antibodies and (E and F) C4 and GT38 antibodies. The tau protein segments recognized by the T13, 4R, and C4 antibodies are specified in parentheses. The undigested tau bands appear primarily at ~60 kD, and the trypsin-digested tau bands at 25 kD. Trypsin cleaves the *N*-terminal unstructured domain of tau, resulting in the absence of signal for the T13 antibody (residues 2–18) as shown in panel D. Trypsin is unable to cleave the fibril core (residues 303–380), thus signals for the 4R antibody (residues 337-368) and the C4 antibody (residues 354-369) are present in the trypsin-resistant band at 25 kD (D and F). The GT38 antibody, which is specific to the AD tau conformation (exact binding epitope unknown), also binds to the trypsin-resistant fibril core observed in al DS and AD cases indicating similar epitope and conformation is present across all samples.

**Supplementary Figure S3**. Electron microscopy images of filaments from sarkosyl-insoluble fractions from the frontal cortex of Down syndrome. (A) Negative stain images from the four cases (indicated) showing fibrils and other background contaminants. (B) Cryo-EM micrograph image showing tau filaments and filaments attributed to amyloid beta. A 2D class average of a small dataset that shows features similar to amyloid beta filaments is shown (inset).

**Supplementary Figure S4**. Cryo–electron microscopy structure analysis. (A) FSC curves of two independently refined half-maps for the PHF (blue) and SF (orange) showing resolution at FSC = 0.143 and corresponding map versus model curves (dashed) and FSC = 0.5 line. (B) Overlay and α-carbon RMSD values for the PHF and SF structures from case 1 (green) compared with published structures (Protein Data Bank: 7NRQ [red] and 7NRS [purple], respectively).

**Supplementary Figure S5.** Final cryo–electron microscopy refined maps of tau PHF and SF from data acquired using GO-AT8 affinity grids to isolate tau filaments. For DS case 2, the PHF structure was solved to 3.0 Å and the SF structure was solved to 3.2 Å resolution (left); for DS case 4, the PHF structure was solved to 5 Å resolution (right).

**Supplementary Figure S6**. Comparison of 2D averages observed for tau filaments from DS case 4. (A) 2D averages comparing a PHF class and one identified to be in an alternate conformation (CTE) [1] based on distinguishing density in the middle the wide portion of the crossover (arrow). (B) 2D averages generated using relion_helix_toolbox by stitching to show full crossover for comparison between the PHF and alternate fold. The central line of density unique to the alternate fold is indicated (arrow).

1. Falcon B, Zivanov J, Zhang W, et al (2019) Novel tau filament fold in chronic traumatic encephalopathy encloses hydrophobic molecules. Nature 568:420–423. https://doi.org/10.1038/s41586-019-1026-5.
